# Testing the Impact of Depressive and Anxiety Features on the Association between Attention-Deficit/Hyperactivity Disorder Symptoms and Academic Performance among University Students: A Mediation Analysis

**DOI:** 10.3390/brainsci12091155

**Published:** 2022-08-30

**Authors:** Ilaria Riboldi, Cristina Crocamo, Tommaso Callovini, Chiara Alessandra Capogrosso, Susanna Piacenti, Angela Calabrese, Susanna Lucini Paioni, Federico Moretti, Francesco Bartoli, Giuseppe Carrà

**Affiliations:** 1 Department of Medicine and Surgery, University of Milano Bicocca, Via Cadore 48, 20900 Monza, Italy; 2 Division of Psychiatry, University College London, Maple House 149, London W1T 7BN, UK

**Keywords:** academic performance, ADHD, anxiety, depression, university students

## Abstract

Attention deficit/hyperactivity disorder (ADHD) is associated with poor academic performance also among university students. This relationship may be made more complex by comorbid conditions. The aim of this study was to evaluate the mediating role of anxiety and depressive symptoms in the relationship between ADHD and academic performance. Data were drawn from the CAMPUS study (registration number: 0058642/21), an ongoing survey on university students’ mental health. Using a logit model, mediation analyses were carried out to test whether the relationship between ADHD symptoms (assessed by ASRS-5) and academic performance might be mediated by depressive (assessed by PHQ-9) and anxiety (assessed by GAD-7) symptoms. Our results showed that worse academic performance is associated with ADHD symptoms (*p* < 0.001). However, about 24% of the overall association between ADHD symptoms and academic performance was mediated by depressive symptoms (indirect effect: 0.065, 95%CI 0.022; 0.100), whereas the contribution of anxiety symptoms to the model was not significant. Along with the association between ADHD symptoms and poor academic performance, our findings highlight the key mediating role of depressive symptoms, which may be targeted with tailored support, ultimately improving both the academic performance and the well-being of university students with ADHD.

## 1. Introduction

Attention-deficit/hyperactivity disorder (ADHD) has been traditionally thought of as mainly a childhood disorder, with the symptoms of inattention, hyperactivity, and impulsivity tempered in adult age. However, these symptoms persist in up to 60% of cases [1,2], making adult ADHD a prevalent disorder affecting 2.5% (95%CI 2.1–3.1) of the general population worldwide [3]. In addition, high rates of comorbid conditions, with a marked impact on the quality of life, are associated with ADHD [1,4], leading to poor functioning across several domains, including academic achievement [5]. Unfortunately, evidence on the academic performance of young adults with ADHD in higher education is much less consistent than on school performance among children and adolescents [6,7,8]. Indeed, college students with ADHD are more likely to withdraw from classes than their non-ADHD counterparts, have poorer study habits and experience difficulties completing tests and tasks on time [5]. Later on, despite the fact that university students with ADHD could be considered a high-functioning subset among the ADHD clinical population, the relevant symptoms seem correlated with lower grade point averages (GPAs), more academic difficulties [9], and slower progress in academic programs [10]. More importantly, an understanding of the relationship between ADHD and academic performance is made still more complex by the high prevalence of comorbid mental health conditions [11,12], including depressive and anxiety symptoms, with prevalence rates in adults with ADHD being around 38% and 47%, respectively [13]. However, both these clinical conditions are supposed to be independently associated with worse academic performance. It has been shown that individuals experiencing depression are more likely to miss classes, tests, and assignments, and to drop courses [14,15]. Similarly, anxiety symptom scores are associated with poorer academic performance, including lower GPAs [16].

Despite the fact that evidence on the relative impact of ADHD and other common comorbid symptoms on academic performance would have important implications for the mental health of these students [17], there are as yet no studies that have evaluated the role of comorbid depressive and anxiety symptoms in the association between ADHD and lower academic performance. In particular, it could be argued that these comorbid symptoms might at least partially mediate this relationship and might make appropriate candidates for directly targeted psychological interventions.

Thus, we conducted a cross-sectional study based on a large and representative sample of university students, hypothesizing that (i) there would be a negative effect of ADHD and depressive and anxiety symptoms on academic performance, and (ii) the association between ADHD symptoms and low academic performance could be at least partially mediated by co-occurring depressive and anxiety symptoms.

## 2. Materials and Methods

### 2.1. Study Design and Setting

This study followed the “Strengthening the Reporting of Observational Studies in Epidemiology” (STROBE) checklist [18]. Data were drawn from the baseline assessment of the “Caring and Assessing Mental health of student Populations at Unimib and uniSurrey (CAMPUS) study”, a large ongoing survey to longitudinally assess the mental health of university students enrolled at the University of Milano-Bicocca (Italy) and the University of Surrey (Guildford, UK). An online survey was delivered by e-mail to students between March and June 2022. In the present work, we report on students from the University of Milano-Bicocca. The study was approved locally by the Ethics Committee of the University of Milano-Bicocca (registration number: 0058642/21); students older than 18 provided their informed consent online. The students could complete the survey in a private setting and quit the survey at any time. The online platform (LimeSurvey) anonymously tracked both complete and partial responses.

### 2.2. Measures

An extensive battery of instruments was used to collect information, although only those instruments relevant to this study are presented here.

#### 2.2.1. Participant Information

We collected information on age, sex, off-campus status, living conditions, and employment status. Data on the degree course, the years of attendance, satisfaction with average exam grades and degree programs, as well as the amount of time spent studying, were also included on the form. Academic performance was investigated using a self-reported binary indicator, i.e., “to be on track with exams”, compared with “not being on track”.

#### 2.2.2. Clinical Measures

ADHD symptoms were assessed using the Italian version of the Adult ADHD Self-Report Screening Scale for DSM-5 (ASRS-5), a short, validated self-report screening instrument [19,20] that is designed to detect the relevant symptoms in the adult population that possibly deserve further clinical evaluation. This is calibrated with the DSM-5 criteria and includes six structured questions and five response options, i.e., “never”, “rarely”, “sometimes”, “often” and “very often”, with a score ranging from 0 (“never”) to 4 (“very often”), thus resulting in a total score of between 0 and 24.

In terms of depressive symptoms assessment, we used the 9-item Patient Health Questionnaire (PHQ-9) [21,22], the relevant module of the self-administered version of the Primary Care Evaluation of Mental Disorders (PRIME-MD) [23]. It enables screening, diagnosis, and severity assessment through nine items that reflect the symptoms of major depression according to DSM-IV over the last two weeks. Possible answers include “never”, “several days”, “more than half the days”, and “nearly every day” and each item is rated from 0 (“never”) to 3 (“nearly every day”).

The 7-item General Anxiety Disorder scale (GAD-7) [24] was used to assess the symptoms of generalized anxiety disorder; participants were asked to report how often they experienced the relevant symptoms over the previous two weeks. Response options include “never”, “some days”, “more than half the days”, and “almost every day”, with scores of 0, 1, 2, and 3, respectively.

### 2.3. Statistics

Statistical analyses were run using the Stata program (version 17, StataCorp. 2021. Stata Statistical Software, College Station, TX, USA). After performing validity and consistency checks of the collected data, descriptive analyses were carried out to summarize the characteristics of the survey participants. In addition, we addressed the missing data issues, verifying that information on ADHD symptoms was not available when the participants were early quitters of the study (i.e., unanswered items > 40%). We compared those students who completed the survey to the early quitters, in order to identify the characteristics that might potentially account for differences in the likelihood of completing the survey. Therefore, assuming that non-response depends, at most, on covariates, we ruled out a “missing not at random” (MNAR) condition.

Standard statistics were provided for both continuous and categorical variables, stratifying them by on-track student status, using the binary indicator as a proxy measure of academic performance. Potential differences in academic performance were thus assessed across individual characteristics by running both Pearson’s chi-square test for categorical variables and performing parametric (e.g., Student’s *t*-test or an analysis of variance (ANOVA)) or non-parametric (e.g., Wilcoxon–Mann–Whitney test) analyses for continuous variables, according to potential deviations of relevant assumptions (e.g., normally distributed residuals). We provided relevant effect sizes via Cohen’s d for each clinical feature. Then, using a binary logistic (logit) model [25], we carried out mediation analyses to test whether the relationship between ADHD symptoms (as an independent variable) and academic performance (as a dependent variable) was direct or whether putative mediator variables would account for the relationship between them (i.e., depressive and anxiety symptoms, as assessed by PHQ-9 and the GAD-7 scale, respectively). Each putative mediator was entered in separate models to assess their individual impact on this relationship. All models were adjusted for sex, age, satisfaction with the degree program, and individual living conditions, potentially accounting for differences in the likelihood of completing the survey. Regression coefficients with 95% confidence intervals (95%CI) were reported. Similarly, estimates for the direct, indirect, and total effects were provided. Statistical significance was set at a *p*-value of 0.05. The flowchart of data analysis is shown in Figure S1 in the Supplementary Materials.

## 3. Results

### 3.1. Sample Characteristics

The online survey was delivered to all university students older than 18, gathering a total of 2134 responses. As a whole, 91% of the sample reported information regarding ADHD symptoms. Therefore, the final sample comprised 1943 undergraduate and postgraduate students, including 425 (22%) men and 1469 (76%) women, with a mean age (standard deviation, SD) of 22.9 (5.0) years. Most students (64%) were attending the first two years of their academic career, mainly enrolled in life sciences (481/25%), psychology (350/18%), and educational sciences (315/16%) programs. Overall, 1006 (52%) respondents self-reported that they were on track with their studies. Descriptive statistics for those students who completed the survey are summarized in Table 1. Compared to students who left the survey early and did not report information on ADHD symptoms (N = 191), the survey completers (N = 1943) were more likely to be off-campus students (33% vs. 25%; *p* = 0.037) and to live in small areas, rather than in the metropolitan area (66% vs. 56%; *p* = 0.018).

### 3.2. Clinical Assessment and Academic Performance

The ASRS-5 mean score was 9.70 (SD = 3.85) in the whole sample, and poor academic performance was associated with self-reported ADHD symptoms (*p* < 0.001; d = 0.51). The average score for depressive symptoms, as assessed by PHQ-9, was 9.88 (SD = 6.15). We uncovered an association between depressive symptoms and poor academic performance. Students who were not on track with their programmes scored higher in the PHQ-9 (mean = 11.30, SD = 6.23) than their on-track counterparts (mean = 8.56, SD = 5.77, *p* < 0.001), with a medium effect size (d = 0.46). Anxiety symptoms showed a total mean score of 10.51 (SD = 5.03), as measured by GAD-7. However, participants with regular academic achievements reported lower GAD-7 scores (mean = 9.88, SD = 4.99) compared with those not on track with their studies (mean = 11.20, SD = 4.98; *p* < 0.001; d = 0.26).

#### 3.2.1. Depressive Symptoms: Mediation Analysis (M1)

By controlling for sex, age, satisfaction with the degree program, off-campus status, and living conditions (small areas vs. metropolitan areas), we assessed the joint contribution of ADHD and depressive symptoms to academic performance, exploring the relevant associations (Table 2). We tested depressive symptoms as a potential mediator of the relationship between ADHD symptoms and the academic performance indicator. A statistically significant direct effect of ADHD symptoms was found regarding academic performance (direct effect: 0.209, 95%CI 0.140; 0.270). However, about 24% of the overall association was mediated by depressive symptoms, as measured by PHQ-9 (indirect effect: 0.065, 95%CI 0.022; 0.100). The results are presented in Table 2.

#### 3.2.2. Anxiety Symptoms: Mediation Analysis (M2)

We also tested the mediating effect of anxiety symptoms on the relationship between ADHD and academic performance (Table 2). Based on the ASRS-5 scores, a statistically significant effect of ADHD symptoms on the likelihood of worse academic achievements was detected (β = 0.137, 95%CI 0.106; 0.168; *p* < 0.001), although the contribution of anxiety symptoms to the model was not significant (*p* = 0.788). Consistently, the indirect effect via anxiety symptoms was negligible and not significant (indirect effect: −0.004, 95%CI −0.033; 0.029). Virtually all the effects of ADHD symptoms on academic performance were direct (direct effect: 0.277, 95%CI 0.216; 0.335).

**Figure 1:**
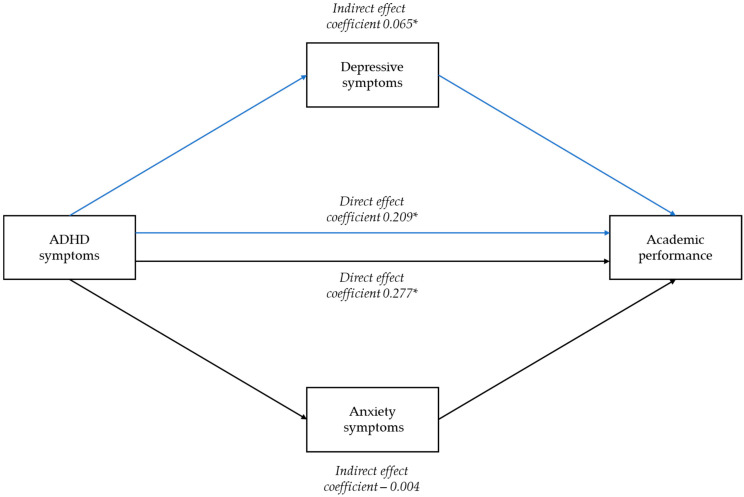
Path diagram of the mediation models between ADHD symptoms and academic performance (being on track vs. not being on track with exams). * *p* < 0.05.

The mediation models are shown in Figure 1.

## 4. Discussion

To our knowledge, this is the first study testing whether the relationship between ADHD symptoms and academic performance in university students can be mediated by symptoms of depression and anxiety. We consistently found an association between ADHD and poor academic performance, which was indeed partially mediated by depressive symptoms, although this was not by anxiety. It is likely that university students with ADHD inherently experience difficulties in academic performance, possibly due to issues related to organization, planning, and time management skills [26,27]. Indeed, the relevant ADHD symptoms seem mainly related to poor study habits [5], lower GPAs [9], and slower progress in academic programs [10].

However, our study also shows the partial mediating role of depressive symptoms on academic performance, which certainly deserves appropriate consideration in terms of research and clinical implications.

Certainly, depression can affect academic performance through a number of mechanisms, with an impact on the attendance of classes and accomplishing tasks, possibly leading students to drop out of courses [14,15]. Depressive symptoms might reduce working memory, which is mainly responsible for planning, strategic choices, and thinking [28], thereby affecting attention distribution [29]. Besides its negative effect on cognitive functioning [30], young people with depression may also find social interaction difficult, enacting avoidance behaviors, with the consequent depletion of their engagement with university courses [31]. In addition, it has been suggested that decreased emotion-regulation ability, known to be associated with depressive symptoms [32], is a key component of individual academic well-being [33]. Finally, depression may further disrupt the multitude of processes involved in ADHD (e.g., attentional control, reward-processing, motivational states, and behavioral inhibition) that influence the ability to regulate emotions and to adapt to external circumstances [34].

However, the interplay between ADHD and depression symptoms in a special population, such as university students, may possibly follow unexpected paths.

ADHD symptoms may be described as a consequence of the alternated balance between “top-down” and “bottom-up” attention processes [35]. Both are impaired in young adults with ADHD, generating inattention, distractibility, and poor frustration tolerance, which may all lead to the dropping of demanding tasks [36,37] and consistently experiencing depressive conditions [38]. Indeed, emotional dysregulation has been proposed as an important intermediate psychological risk factor for ADHD-depression comorbidity in young people [39]. Unfortunately, the deliberate modulation of emotional states and subsequent behaviors, which involves the ability to purposely focus and shift attention, as well as to inhibit or activate specific behaviors as appropriate [40,41,42], is crucial for academic performance.

In addition, the potential role of avoidant behaviors and their connections with both poor academic performance and depressive symptoms should be considered [43,44]. In young adults with ADHD, avoidant behaviors, in terms of both self-isolation [45] and challenging task procrastination [46], are frequent maladaptive strategies that also maintain and worsen depressive symptoms [47,48]. Thus, treatment targets for university students with comorbid ADHD who show internalizing symptoms, such as depression, should address procrastination and avoidant behaviors [49] through strategies improving emotion regulation [50]. Moreover, a timely screening for depression might be considered for subjects with ADHD symptoms who report persisting academic difficulties, to ensure a comprehensive assessment of students’ mental health [51,52].

Our findings should be interpreted with caution, considering some of the methodological limitations. First, the cross-sectional nature of the study does not allow us to draw conclusions regarding the causal relationships between ADHD symptoms, the dependent variable, and any mediating factors, including depression. Consistently, different directional paths might underlie these relationships. In addition, we assessed academic performance using a rough proxy measurement of school achievements, while ADHD symptoms were measured by a brief 6-item scale. Thus, future studies would benefit from more comprehensive and structured diagnostic reporting of ADHD, in order to reduce recall bias and/or under-reporting, as well as potential misclassifications. In addition, our approach, which was based on an online survey to collect clinical information, inevitably involves a number of limitations, including sampling issues [53]. Nonetheless, the survey was held in a private setting, which has been shown to minimize the likelihood of under-reporting and to reduce bias, offering perceived privacy and anonymity, thereby addressing potential stigmatization or embarrassment [54,55]. In addition, it should be considered that the selected participants might be not fully representative of the university students’ population of reference. The respondents are likely to be students who are already engaged and interested in the topic of mental health, which possibly suggests a selection bias.

## 5. Conclusions

Our findings support the well-established association between ADHD symptoms and poor academic performance. In addition, the key mediating role of depressive symptoms in this association was highlighted. Therefore, students with ADHD who report persisting academic problems might also benefit from depression screening, and vice versa. Addressing the special treatment needs of vulnerable university students with ADHD by offering timely and tailored support might ultimately improve their academic performance.

## Figures and Tables

**Table 1:** Sociodemographic and clinical characteristics distribution according to academic performance (being on track vs. not being on track with exams).

Characteristics	Total	On Track with Exams	Not on Track	*p*-Value
	N = 1943	N = 1006 (52%)	N = 937 (48%)	
	*N* (*%*) *or Mean* (*SD*)			
***Sex***				0.850 ^a^
Women	1469 (76%)	765 (76%)	704 (75%)	
Men	425 (22%)	215 (21%)	210 (22%)	
***Age*, *years***	22.93 (5.00)	22.50 (4.70)	23.39 (5.27)	<0.001 ^c^
***Off-campus status***				0.699 ^a^
Off-campus	647 (33%)	339 (34%)	308 (33%)	
Local	1296 (67%)	667 (66%)	629 (67%)	
***Employment***				<0.001 ^a^
Non-worker	1018 (52%)	573 (57%)	445 (47%)	
Temporary worker	471 (24%)	237 (24%)	234 (25%)	
Part-time or full-time worker	454 (23%)	196 (19%)	258 (27%)	
***Satisfaction with exam grades***				<0.001 ^a^
Very unsatisfied	76 (4%)	9 (1%)	67 (7%)	
Unsatisfied	236 (12%)	65 (6%)	171 (18%)	
Moderately satisfied	527 (27%)	215 (21%)	312 (33%)	
Satisfied	688 (35%)	443 (44%)	245 (26%)	
Very satisfied	346 (18%)	260 (26%)	86 (9%)	
***Satisfaction with the degree program***				<0.001 ^a^
Very unsatisfied	50 (3%)	14 (1%)	36 (4%)	
Unsatisfied	114 (6%)	49 (5%)	65 (7%)	
Moderately satisfied	347 (18%)	133 (13%)	214 (23%)	
Satisfied	747 (38%)	381 (38%)	366 (39%)	
Very satisfied	663 (34%)	418 (42%)	245 (26%)	
***Time spent studying***				<0.001 ^a^
>5 h	524 (27%)	322 (32%)	202 (22%)	
From 3 to 5 h	686 (35%)	372 (37%)	314 (33%)	
From 1 to 3 h	552 (28%)	265 (26%)	287 (31%)	
<1 h	122 (6%)	34 (3%)	88 (9%)	
***ADHD symptoms (ASRS-5)***	9.70 (3.85)	8.77 (3.77)	10.69 (3.70)	<0.001 ^b^
***Depressive symptoms (PHQ-9)***	9.88 (6.15)	8.56 (5.77)	11.30 (6.23)	<0.001 ^b^
***Anxiety symptoms (GAD-7)***	10.51 (5.03)	9.88 (4.99)	11.20 (4.98)	<0.001 ^c^

^a^ Pearson’s chi-square test; ^b^ Student’s *t*-test; ^c^ Wilcoxon–Mann–Whitney test.

**Table 2:** A test of the mediation between ADHD symptoms and the academic performance indicator (being on track vs. not being on track with exams): depressive and anxiety symptoms, as assessed by PHQ-9 and GAD-7, respectively *.

Term	Coefficient	Standard Error	95% CI	OR	*p*-Value
Depressive symptoms (M1) ^β^	0.036	0.011	0.015; 0.056	1.036	0.001
ADHD symptoms ^β^	0.104	0.017	0.070; 0.138	1.110	<0.001
***Test of mediation***					
Indirect effect	0.065	0.020	0.022; 0.100		
Direct effect	0.209	0.034	0.140; 0.270		
Total effect	0.274	0.028	0.217; 0.330		
**Term**	**Coefficient**	**Standard error**	**95%CI**	**OR**	***p*-Value**
Anxiety symptoms (M2) ^β^	−0.003	0.012	−0.026; 0.020	0.997	0.788
ADHD symptoms ^β^	0.137	0.016	0.106; 0.168	1.147	<0.001
***Test of mediation***					
Indirect effect	−0.004	0.016	−0.033; 0.029		
Direct effect	0.277	0.031	0.216; 0.335		
Total effect	0.273	0.026	0.225; 0.319		

* Controlling for sex, age, degree course satisfaction, off-campus status, and living area (overall significance of *p* < 0.001). ^β^ Beta coefficient and the related estimates for the association of the selected independent variable with academic performance; OR: odds ratio = exp (beta coefficient).

## Data Availability

Not applicable.

## Supplementary Materials

The following supporting information can be downloaded at: https://www.mdpi.com/article/10.3390/brainsci12091155/s1, Figure S1: Flowchart of data analysis.
