# Supplementary material for: Testing the Impact of Depressive and Anxiety Features on the Association between Attention-Deficit/Hyperactivity Disorder Symptoms and Academic Performance among University Students: A Mediation Analysis
Source: Brain Sci. 2022 Aug 30;12(9):1155. doi: 10.3390/brainsci12091155 (PMC9496751; doi:10.3390/brainsci12091155)
Supplement: Supplementary file 1 [file brainsci-12-01155-s001.zip › brainsci-1874529-supplementary.pdf]

Figure S1. Flowchart of data analysis.

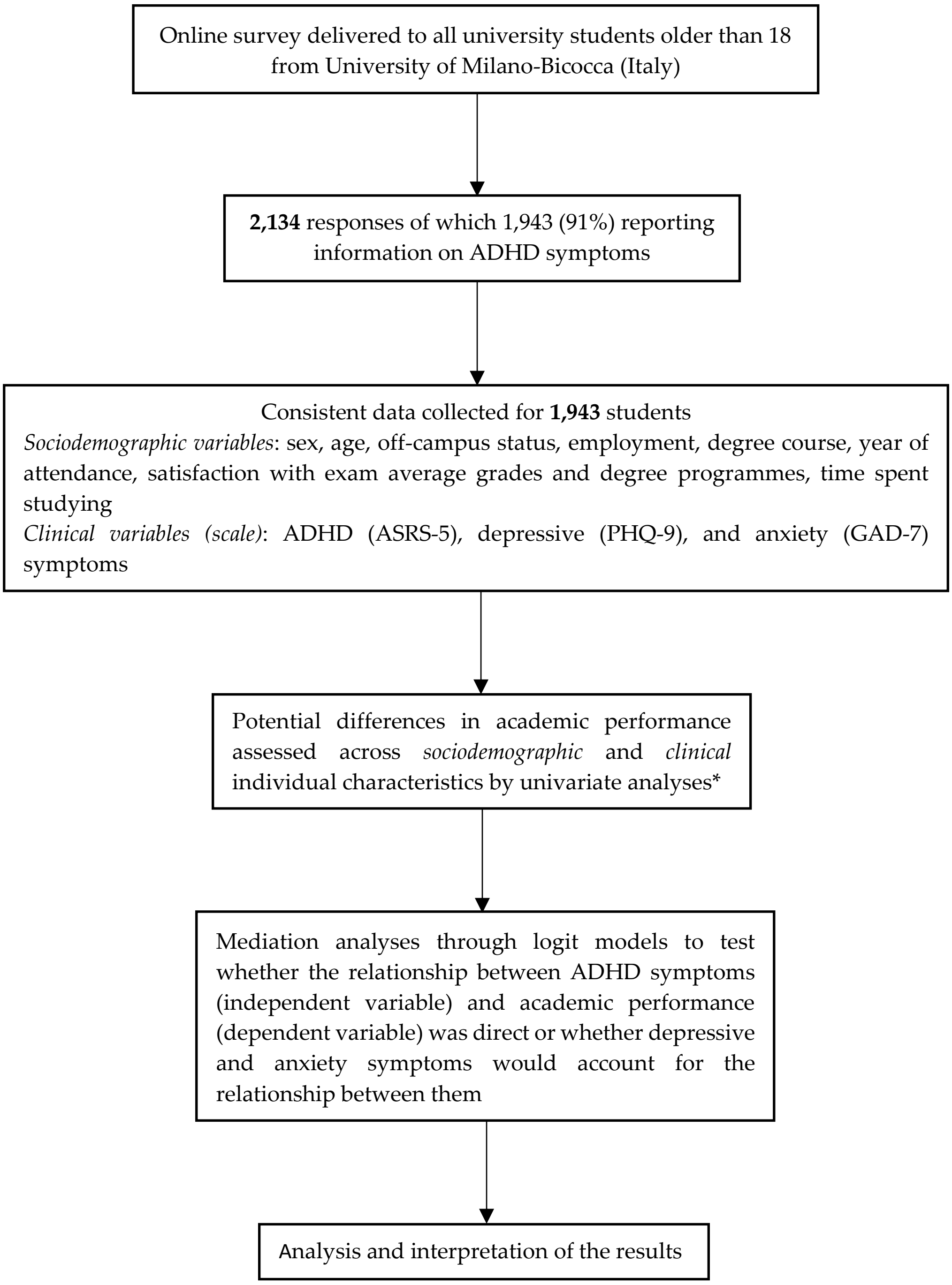

* Pearson's chi-square test for categorical variables and parametric (e.g., Student's t test, analysis of variance) or non-parametric (e.g., Wilcoxon-Mann-Whitney test) analyses for continuous variables
